# Feasibility of using death certificates for studying place of death in Latin America

**DOI:** 10.26633/RPSP.2021.149

**Published:** 2021-12-16

**Authors:** Katja Seitz, Luc Deliens, Joachim Cohen, Emanuel Adrian Cardozo, Vilma A. Tripodoro, Fernando Cesar Iwamoto Marcucci, Luís Fernando Rodrigues, Lea Derio, Miguel Antonio Sánchez-Cárdenas, Valentina Salazar, Victor Rolando Samayoa, Ximena Pozo, Diane A. Dykeman-Sabado, Celina Castañeda de la Lanza, Nineth Carolina Baltodano Algaba, Gabriela Píriz Alvarez, Leticia Viana, Tulio González, Tania Pastrana

**Affiliations:** 1 Department of Palliative Medicine, Medical Faculty, RWTH Aachen University Department of Palliative Medicine, Medical Faculty, RWTH Aachen University Aachen Germany; 2 End-of-Life Care Research Group, Vrije Universiteit Brussel and Ghent University End-of-Life Care Research Group, Vrije Universiteit Brussel and Ghent University Brussels Belgium; 3 National Ministry of Health National Ministry of Health Buenos Aires Argentina; 4 Department of Palliative Care, Institute of Medical Research A. Lanari, University of Buenos Aires Department of Palliative Care, Institute of Medical Research A. Lanari, University of Buenos Aires Buenos Aires Argentina; 5 Hospital Dr. Anísio Figueiredo, State Health Secretariat of Paraná Hospital Dr. Anísio Figueiredo, State Health Secretariat of Paraná Londrina Brazil; 6 Palliative Care Unit, Barretos Cancer Hospital Palliative Care Unit, Barretos Cancer Hospital Barretos Brazil; 7 Nursing Department, Faculty of Medicine, University of Chile Nursing Department, Faculty of Medicine, University of Chile Santiago Chile; 8 ATLANTES Global Palliative Care Observatory, Institute for Culture and Society, University of Navarra ATLANTES Global Palliative Care Observatory, Institute for Culture and Society, University of Navarra Pamplona Spain; 9 Faculty of Health Sciences, University of Tolima Faculty of Health Sciences, University of Tolima Ibague Colombia; 10 Palliative Care Unit, Institute of Cancerology Palliative Care Unit, Institute of Cancerology Guatemala City Guatemala; 11 Palliative Care Unit, Hospital Comprehensive Care for the Elderly, Ministry of Public Health Palliative Care Unit, Hospital Comprehensive Care for the Elderly, Ministry of Public Health Quito Ecuador; 12 Foundation Corazon del Siervo Foundation Corazon del Siervo Santo Domingo Dominican Republic; 13 Coordination for Advance Directives and Palliative Care Program, Institute of Health of the State of Mexico, Ministry of Health of Mexico Coordination for Advance Directives and Palliative Care Program, Institute of Health of the State of Mexico, Ministry of Health of Mexico Toluca Mexico; 14 Oncology Center for Chemotherapy and Palliative Care, Ministry of Health Oncology Center for Chemotherapy and Palliative Care, Ministry of Health Managua Nicaragua; 15 Faculty of Medicine, University of the Republic Faculty of Medicine, University of the Republic Montevideo Uruguay; 16 Department of Palliative Care and Pain, National Cancer Institute Department of Palliative Care and Pain, National Cancer Institute Capiata Paraguay; 17 Institute of Oncology Dr. Luis Razetti Institute of Oncology Dr. Luis Razetti Caracas Venezuela

**Keywords:** Death certificates, comparative study, Latin America, Certificado de defunción, estudio comparativo, América Latina, Atestado de óbito, estudo comparativo, América Latina

## Abstract

**Objective.:**

This paper assesses the availability and quality of death certificate data in Latin America and the feasibility of using these data to study place of death and associated factors.

**Methods.:**

In this comparative study, we collected examples of current official death certificates and digital data files containing information about all deaths that occurred during 1 year in 19 Latin American countries. Data were collected from June 2019 to May 2020. The records for place of death and associated variables were studied. The criteria for data quality were completeness, number of ill-defined causes of death and timeliness.

**Results.:**

All 19 countries provided copies of current official death certificates and 18 of these registered the place of death. Distinguishing among hospital or other health care institution, home and other was possible for all countries. Digital data files with death certificate data were available from 12 countries and 1 region. Three countries had data considered to be of high quality and seven had data considered to be of medium quality. Categories for place of death and most of the predetermined factors possibly associated with place of death were included in the data files.

**Conclusions.:**

The quality of data sets was rated medium to high in 10 countries. Hence, death certificate data make it feasible to conduct an international comparative study on place of death and the associated factors in Latin America.

## RESUMEN

**Objetivo.:**

En este artículo se evalúa la disponibilidad y la calidad de los datos del certificado de defunción en América Latina y la factibilidad de emplear estos datos para estudiar el lugar de defunción y factores asociados.

**Métodos.:**

En este estudio comparativo, se recogieron ejemplos de certificados oficiales de defunción actuales y archivos digitales de datos que contenían información acerca de todas las defunciones que ocurrieron durante un año en 19 países latinoamericanos. Se recopilaron datos desde junio del 2019 hasta mayo del 2020. Se estudiaron los registros del lugar de la muerte y las variables asociadas. Los criterios para determinar la calidad de los datos fueron la exhaustividad, el número de causas de muerte mal definidas y la presentación oportuna de la información.

**Resultados.:**

Los 19 países proporcionaron copias de los certificados oficiales de defunción actuales; en 18 de estos se registraba el lugar de la muerte. En todos los países fue posible distinguir entre hospital u otra institución de atención de salud, el hogar y otros. Se obtuvieron los archivos de datos digitales con los datos del certificado de defunción de 12 países y una región. Tres países tenían datos considerados de buena calidad y siete tenían datos considerados de calidad media. En los archivos de datos se incluyeron categorías para lugar de defunción y la mayoría de los factores predeterminados posiblemente asociados con el lugar de defunción.

**Conclusiones.:**

La calidad de los conjuntos de datos se calificó de media a buena en 10 países. En consecuencia, es factible realizar un estudio internacional comparativo sobre el lugar de defunción y los factores asociados en América Latina con los datos del certificado de defunción.

## RESUMO

**Objetivo.:**

Este estudo avalia a disponibilidade e a qualidade dos dados das declarações de óbito na América Latina e a viabilidade de usar esses dados para estudar o local do óbito e fatores associados.

**Métodos.:**

Neste estudo comparativo, coletamos exemplos de declarações de óbito oficiais atuais e arquivos de dados digitais contendo informações sobre todos os óbitos que ocorreram durante 1 ano em 19 países latino- americanos. Os dados foram coletados no período de junho de 2019 a maio de 2020. Foram estudados os registros do local do óbito e variáveis associadas. Os critérios de qualidade dos dados foram preenchimento completo, número de causas mal definidas de morte e oportunidade.

**Resultados.:**

Todos os 19 países forneceram cópias das declarações de óbito oficiais atuais, e 18 deles registraram o local do óbito . Foi possível distinguir em todos os países entre hospital ou outra instituição de saúde, lar ou outro local. Arquivos de dados digitais com os dados das declarações de óbito foram disponibilizados por 12 países e 1 região. Três países tiveram dados considerados de alta qualidade, e sete tiveram dados considerados de qualidade média. As categorias de local do óbito e a maioria dos fatores predeterminados possivelmente associados ao local do óbito foram incluídos nos arquivos de dados.

**Conclusões.:**

A qualidade dos conjuntos de dados foi classificada como média a alta em 10 países. Portanto, os dados de declarações de óbito possibilitam a realização de um estudo comparativo internacional sobre local do óbito e fatores associados na América Latina.

Knowing where people die is crucial for health care planning and for enabling patients to receive adequate care at their preferred place of death (1, 2). Due to the epidemiological and demographic transitions in Latin America that are leading to more people with chronic illness, an increasing need for end-of-life care is expected (3). In order to establish functioning care services and ensure they are available, it is necessary to identify locations where services and resources should be assigned (1). In addition to the benefits for patients and their caregivers, improving services for end-of-life care can provide economic advantages for the system (4). However, in Latin America only nine studies on the place of death have taken place: five in Mexico (5-9), two in Brazil (10, 11) and two in Chile (12, 13). None of these studies included the entire population of a country or compared results to other countries in the region.

Vital statistics can provide answers to the questions of when, where and how people die. Ideally, they derive from data collected by civil registration, such as death certificates (14). Well-maintained data drawn from civil registrations are a comprehensive and reliable resource for research and development of health care services (15, 16). Several approaches have been used for evaluating the quality and availability of vital statistics, civil registration systems and death certificate data in Europe and globally (17-21). Previous studies in Europe have shown the value of using death certificates for studying place of death (17).

In this paper, we assess the availability and the quality of death certificate data for 1 year in 19 Latin American countries and the feasibility of using these data to study place of death and associated factors.

## METHODS

### Study design

In this first comparative study of the feasibility of using data from death certificates to study the place of death, we collected examples of current official death certificates and digital data files containing information about all deaths for the most recent year available in 19 Latin American countries: Argentina, Bolivia (Plurinational State of), Brazil, Chile, Colombia, Costa Rica, Cuba, Dominican Republic, Ecuador, El Salvador, Guatemala, Honduras, Mexico, Nicaragua, Panama, Paraguay, Peru, Uruguay and Venezuela (Bolivarian Republic of). The data were collected from June 2019 to May 2020.

A common database was created from the data files. Variables such as place of death, cause of death and other sociodemographic variables identified as relevant in the literature (17, 22, 23) were extracted (Table 1).

### Availability of data

To obtain the data, at least one research partner in every country was contacted and formally invited to collaborate on the project. Selection criteria for research partners were: (i) having expertise in conducting research, (ii) having access to the national information system and (iii) having experience with collaboration. The research partners are coauthors of this paper.

Research partners were asked to send a copy of the death certificate used in their country and to complete a questionnaire about the data files available and the prerequisites for obtaining them.

**TABLE 1.** Information from data files about deaths in 19 Latin American countries included in the database

Characteristic	Description
Year of registration or death^a^	Most recent year available
Study population	All deaths in the country or region
	Place of death (hospital, home, other health care institution, other)
	Cause of death (ICD code)
Variables	Age
	Sex
	Civil status
	Level of education
	Municipality of death
	Municipality of residence
	Urban or rural area

ICD: *International Statistical Classification of Diseases and Related Health Problems*.

a Either the year of death or year of registration was used depending on the country.

***Source:*** Table prepared by the authors.

Countries that submitted a copy of the official death certificate and/or a data file derived from death certificate data (with place of death information) were included.

### Feasibility of using data

The data files and death certificates from each country were reviewed. To evaluate the feasibility of studying place of death and associated factors, the content and quality were analyzed, considering the following characteristics: (i) the content of the data, including the most recent year for which data were available, the total number of deaths in the data file, information on place of death and the availability of information about other variables possibly associated with place of death; and (ii) the quality of the data, which was assessed through indicators including completeness – that is, aiming for 100% registration, with the rate of mortality underregistration provided by the Pan American Health Organization (PAHO) (24) – and the quality and timeliness of reporting.

No indicator to estimate the quality of reporting of place of death was identified, so we used the indirect indicator of “quality of cause of death,” described by Mathers et al. (18). This method classifies the data as being of high, medium or low quality based on the proportion of ill-defined codes (that is, underlying causes of death that give nonspecific information and lack diagnostic meaning, modified from Mathers et al.) (18) and the percentage of completeness (18, 20, 21). Those records that were more than 90% complete and had a percentage of ill-defined causes of death below 10% were considered to be of high quality, those with completeness between 70% and 90% or with the percentage of ill-defined causes of death between 10% and 20% were categorized as medium quality, and those with completeness below 70% or with the percentage of ill-defined codes above 20% were classified as low quality (18).

Timeliness was evaluated according to the World Health Organization’s (WHO’s) assessment tool (21), which rates registration systems as being highly adequate when data take less than 3 years to be published. A duration of 3 years is considered adequate.

### Statement of ethical approval

The project was approved by the ethics committee of RWTH Aachen University (approval no. EK 206/19); no additional approvals were needed from local ethics committees in Latin America. The data collected were completely anonymous, so no personal identification was possible.

## RESULTS

### Availability of death certificates

Altogether 19 countries provided a copy of their official death certificate (Table 2). In Argentina, every province has its own death certificate. For our study, we obtained the one used in Buenos Aires City. We excluded Bolivia (Plurinational State of) from the analysis because the place of death is registered only in cases of nonnatural death.

### Availability of data files

Digital data files with death certificate data including information about the place of death were obtained from 12 countries (Argentina, Brazil, Chile, Colombia, Costa Rica, Ecuador, El Salvador, Guatemala, Mexico, Paraguay, Peru and Uruguay) and one region (Caracas, Bolivarian Republic of Venezuela). No data files were provided by Cuba, the Dominican Republic, Honduras, Nicaragua and Panama (Table 2).

**TABLE 2.** Availability of death certificates and data files in 19 Latin American countries, by country

Country	Data availability	Most recent year of death or registration available in data file^a^	Total number of deaths in data file	Total population^b^	Death rate^c^	Comments
**Argentina**	Death certificate (Buenos Aires City) and data file	2017	341 688	44 044 811	0.78	Via research partner; death certificate different for every region
**Bolivia (Plurinational State of)**	Death certificate	NA	NA	NA	NA	No place of death reported on death certificate
**Brazil**	Death certificate and data file	2017	1 312 663	207 833 831	0.63	Via research partner
**Chile**	Death certificate and data file	2016	104 026	18 209 068	0.57	Online; death certificate via research partner
**Colombia**	Death certificate and data file	2017	227 624	48 909 844	0.47	Online; individual data on age were requested separately
**Costa Rica**	Death certificate and data file	2016	22 601	4 899 345	0.46	Online, after registration; dictionary via research partner
**Cuba**	Death certificate	NA	NA	NA	NA	Via research partner
**Ecuador**	Death certificate and data file	2017	70 841	16 785 361	0.42	Online
**El Salvador**	Death certificate and data file	2017	39 602	6 388 122	0.62	Via research partner
**Guatemala**	Death certificate and data file	2017	81 726	16 087 418	0.51	Via research partner
**Honduras**	Death certificate	NA	NA	NA	NA	Via research partner; data not digitized, according to research partner
**Mexico**	Death certificate and data file	2017	703 047	124 777 324	0.56	Online
**Nicaragua**	Death certificate	NA	NA	NA	NA	Via research partner
**Panama**	Death certificate	NA	NA	NA	NA	Via research partner
**Paraguay**	Death certificate and data file	2017	29 021	6 867 062	0.42	Via research partner
**Peru**	Death certificate and data file	2017	121 142	31 444 297	0.39	Via official request and research partner
**Dominican Republic**	Death certificate	NA	NA	NA	NA	Via research partner
**Uruguay**	Death certificate and data file	2018	34 128	3 449 299	0.99	Via research partner
**Venezuela (Bolivarian Republic of)**	Death certificate and data file (Caracas region)	2016	18 590	No data	NA	Via research partner

NA: not available.

a The year of registration was available for Brazil, Chile, Colombia, Paraguay and Venezuela (Bolivarian Republic of); the year of death was available for Argentina, Costa Rica, Ecuador, El Salvador, Guatemala, Mexico, Peru and Uruguay.

b This category refers to the total population of the country or region during the reference year for the data file.

c This is the number of registered deaths in the data file divided by the total population.

***Source:*** Table prepared by the authors from their data, with the exception of the total population, for which the source was reference 36.

The procedure for obtaining the data files varied. Data files from Chile, Colombia, Ecuador and Mexico were publicly accessible on the homepage of the institution in charge (25-29) and came with corresponding dictionaries for the names of the variables and information about the codes used in the file. Data files and dictionaries were submitted by the research partners for Argentina, Brazil, El Salvador, Guatemala, Paraguay, Uruguay and Venezuela (Bolivarian Republic of).

In Costa Rica (30), Peru and Uruguay, it was necessary to make additional official requests. No fees had to be paid in any of the countries to obtain the data. The process of obtaining the data files took between 3 weeks (Paraguay) and 12 months (Peru).

### Feasibility of studying place of death

**Most recent year available and total number of deaths.** At the time of data collection, data files were available from three countries for 2016, from eight countries for 2017 and from one country for 2018 (Table 2). The total number of deaths in the population ranged from 22 601 in Costa Rica to 1 312 663 in Brazil, excluding stillbirths. In the region of Caracas, Venezuela (Bolivarian Republic of), the total number of deaths was 18 590 (Table 2).

**Place of death information on death certificates and in data files.** In every country, death at home was included on the death certificate and a distinction could be made between death at a hospital or other health care institution and at home or other (Table 3). Distinguishing between hospital and other health care institution was possible only in eight countries.

In Argentina, Costa Rica, Dominican Republic, Ecuador, Honduras, Mexico, Nicaragua, Paraguay and Peru, the category “hospital” was not available on the death certificate. For example, in Costa Rica and Panama, the place of death was written rather than coded, so categories could be added to the database created for this study. In Argentina, Ecuador, Mexico, Paraguay and Peru, the health care provider was reported. In Latin America, different providers operate their own facilities within the health care system. Because most providers operate only hospitals, these facilities were recoded into the category “hospital” in our database.

The categories “workplace” (4 countries) and “public place” (10 countries) are also common on death certificates (Table 3).

In most countries, the categories for place of death in the data files were identical to the categories on the death certificates. In Costa Rica, the data file contained more categories than the death certificate because written text was recoded. In Ecuador, the categories in the data file were slightly different from those on the death certificate.

**TABLE 3.** Categories for place of death recorded on death certificates and in data files in 18 countries in Latin America, by country

Country or region^a^	Place of death					Percentage of data files missing information about place of death
	Hospital	Other health care institution	Home	Public place	Other	
**Both death certificate and data file available**						
Argentina	Institutions combined	Institutions combined	Yes	NA	Yes^b^	1.3
Brazil	Yes	Yes	Yes	Yes	Yes	0.1
Chile	Yes	NA	Yes	NA	Yes	0.0
Colombia	Yes	Yes	Yes	Yes	Yes, workplace	0.7
Costa Rica	Text only^c^	Text only^c^	Yes	Yes	Only from data file	0.3
Ecuador	Institutions combined	Institutions combined	Yes	NA	Yes	0.0
El Salvador	Yes	Yes	Yes	Yes	Yes	0.0
Guatemala	Yes	Yes	Yes	Yes	Yes, workplace	5.8
Mexico	Institutions combined	Institutions combined	Yes	Yes	Yes	2.2
Paraguay	Institutions combined	Institutions combined	Yes	Yes	Yes	0.0
Peru	Institutions combined	Institutions combined	Yes	Yes	Yes, workplace	25.5
Uruguay	Yes	Yes	Yes	Yes	Yes	0.1
Venezuela (Bolivarian Republic of; Caracas region)	Yes	Only from data file	Yes	Yes	Only from data file, workplace	7.1
**Only death certificate available**						
Cuba	Yes	Yes	Yes	NA	Yes	
Dominican Republic	Institutions combined	Institutions combined	Yes	Yes	Yes	
Honduras	Institutions combined	Institutions combined	Yes	NA	Yes	
Nicaragua	Institutions combined	Institutions combined	Yes	NA	Yes	
Panama	Text only	Text only	Text only	Text only	Text only	

NA: Not available

a Bolivia was excluded from the analysis because place of death is registered only for nonnatural deaths.

b This category includes residential care for elderly people.

c Includes one category for each institution or hospital.

***Source:*** Table prepared by the authors from their data.

The rate of deaths registered in the data file without information about the place of death was highest in Peru at 25.5%, and it was between 0.0% and 5.8% in the other countries, but in the region of Caracas it was 7.1% (Table 3).

**Variables potentially associated with place of death.** Sociodemographic variables were available from all countries (Table 4). Age, sex and civil status (single, married, widowed, divorced, separated, civil partnership) were available from every country except for Colombia (where online data provided age only in aggregated form) and Argentina (where civil status was not available). The level of education was provided from every country except Costa Rica and El Salvador. Categories, however, were inconsistent between the countries due to different educational systems.

With the exceptions of Chile, Costa Rica and Ecuador, the location of death and place of residence were available with the precision of what in an international context would correspond to municipality. Zip codes are not commonly used in Latin America.

Chile, Colombia, Costa Rica, Ecuador, Guatemala, Mexico and Venezuela (Bolivarian Republic of) provided information about whether the place of death or residence, or both, could be classified as rural or urban. In Brazil, this information could be gained through linkage to an external file, and in El Salvador through the location of the place of death or residence.

**TABLE 4.** Availability of variables possibly associated with place of death in data files for 13 countries in Latin America, by variable

Variable	Country												
	Argentina	Brazil	Chile	Colombia	Costa Rica	Ecuador	El Salvador	Guatemala	Mexico	Paraguay	Peru	Uruguay	Venezuela (Bolivarian Republic of)
**Sociodemographic variables**													
Age	X	X	X	X^a^	X	X	X^b^	X	X	X	X	X	X
Sex	X	X	X	X	X	X	X	X	X	X	X	X	X
Civil status		X	X	X	X	X	X	X	X	X	X	X	X
Level of education	X	X	X	X		X		X	X	X	X	X	X
**Clinical characteristics**													
Cause of death, including ICD-10 code	X	X	X	X	X	X	X	X	X	X	X	X	X^c^
**Residence characteristics**													
Municipality of death	X	X		X	X	X	X	X	X	X	X	X	X
Municipality of residence	X	X	X	X	X	X^d^	X	X	X	X	X	X	X
Urban or rural place of death		X^e^		X		X	X^f^	X				X^c^	
Urban or rural residence		X^e^	X	X	X	X	X^f^		X			X^c^	

X: data available.

a Only aggregated information available.

b Available only up to age 99.

c Missing in more than 50% of cases.

d Available only for the province, not the municipality.

e Available through linkage to an external file.

f Information could be deduced from the variable “Municipality/canton”.

***Source:*** Table prepared by the authors from their data.

All countries use the *International Statistical Classification of Diseases and Related Health Problems*, 10th edition, (ICD-10) to code the underlying cause of death.

### Quality assessment

**Completeness of registration.** Completeness of mortality registration ranged from 54% in Peru to 100% in Argentina, Mexico and Uruguay (Table 5).

**Quality of reporting.** The proportion of ill-defined codes ranged from 3.8% in Mexico to 30% in El Salvador. In every country, an ICD-10 code was registered for all deaths, except for Caracas, Bolivarian Republic of Venezuela, where cause of death information was missing in 54.6% of deaths in the data file. To avoid distorting the rate of ill-defined codes in relation to all cause of death codes, we added cases with missing cause of death information to the category of ill-defined codes. The databases obtained in this study are considered to be of medium quality (Table 5).

**Timeliness.** Timeliness was considered to be highly adequate for the 13 countries that provided a digital data file, according to the WHO assessment tool (21). Since we received the data in 2019–2020 and the data files refer to the years 2016 (received in 2019), 2017 and 2018 (received in 2019 and 2020), the time from data collection to data publication is less than 3 years for all countries.

## DISCUSSION

All 19 Latin American countries included in this study provided examples of their death certificates: 18 registered the place of death on the certificate and 13 provided a digital data file. None of the data were older than 3 years. For all countries, it was possible to distinguish the place of death among a hospital or other health care institution, home and other. The data are considered to be of high quality for three countries, medium quality for seven countries and low quality for three countries. Different categories for the place of death, as well as most of the predetermined factors possibly associated with it, are available from the data files, making it feasible to conduct an international comparative study of the place of death in Latin America.

**TABLE 5.** Quality assessment of data files for death certificates from 13 countries in Latin America, by country

Country	Completeness of mortality registration^a^(%)	Ill-defined codes^b^(%)	Timeliness of reporting^c^	Quality of data
**Argentina**	100	16.3	Highly adequate	Medium
**Brazil**	98	9.5	Highly adequate	High
**Chile**	93	5.1	Highly adequate	High
**Colombia**	78	4.5	Highly adequate	Medium
**Costa Rica**	86	5.6	Highly adequate	Medium
**Ecuador**	80	10.4	Highly adequate	Medium
**El Salvador**	90	30.0	Highly adequate	Low
**Guatemala**	99	14.0	Highly adequate	Medium
**Mexico**	100	3.8	Highly adequate	High
**Paraguay**	85	7.9	Highly adequate	Medium
**Peru**	54	9.4	Highly adequate	Low
**Uruguay**	100	16.2	Highly adequate	Medium
**Venezuela (Bolivarian Republic of; Caracas region)**	89	66.1^d^	Highly adequate	Low

a Completeness of mortality registration was calculated for each country by subtracting from 100% the rate of mortality underreporting as provided by the Pan American Health Organization’s online database for the health situation in the Americas (24). In each country, the rate for the most recent year available was used.

b This represents the number of deaths assigned to ill-defined codes or without cause of death information divided by the total number of deaths.

c Timeliness was assessed using the WHO assessment tool (21).

d Cause of death information was missing for 54.6% of deaths.

***Source:*** The table was prepared by the authors based on the results of their study, with the exception of the category for completeness.

In general, rather than distinguishing between hospitals and other health care institutions (such as nursing homes or outpatient facilities), a distinction is more often made between private and public institutions (for example, in Argentina, Guatemala, Mexico, Panama, Peru and the Bolivarian Republic of Venezuela) or between different health care providers (for example, in Guatemala, Mexico, Paraguay, Peru). The structure of many health care systems is fragmented and decentralized in Latin America, meaning that they are made up of several main providers within the health care system, with each one operating its own facilities. The affiliation with a particular provider of a facility where the death occurred is primarily documented in the death certificate rather than whether the death occurred in a hospital or another type of facility.

### Availability of data

Retrieving data files in Latin America was relatively straightforward. Although some were available online, most could be obtained within less than 4 months by contacting local research partners and payment was not required. The process seemed to be simpler in Latin America than in European countries, where fees have to be paid and additional approvals are required alongside those provided by the agencies responsible for death certification (17).

### Quality of data

Information on the place of death and most of the sociodemographic variables possibly associated with the place of death were available in the data files.

According to our analysis, Brazil, Chile and Mexico provide mortality data of high quality, while data from Argentina, Colombia, Costa Rica, Ecuador, Guatemala, Paraguay and Uruguay are considered to be of medium quality and data from El Salvador, Peru and Caracas (Bolivarian Republic of Venezuela) were of low quality.

These 13 Latin American countries, including one region, have been included in other studies assessing the quality of global mortality data (18-20). Mathers and colleagues studied completeness and ill-defined codes and whether the most recent ICD version was used (18). Mathers et al. included data from 1990 to 2003, which were the most recent years with at least 50% completeness. They classified data provided by Mexico and the Bolivarian Republic of Venezuela as high quality; data from Ecuador, Paraguay and Peru as low quality; and data from the remaining countries as medium quality (Argentina, Brazil, Chile, Colombia, Costa Rica, El Salvador, Guatemala, Uruguay). European countries performed slightly better in their study. Of the European countries included, almost half were classified as providing medium-quality data, one third as providing high-quality data and less than one fifth as providing low-quality data (18).

In another study, a more comprehensive measure was used, and the average quality of data for the years 2005 to 2009 was analyzed (19). The quality of data was classified as very high in Chile, Costa Rica, Mexico and Venezuela (Bolivarian Republic of); as high in Argentina, Brazil, Colombia, El Salvador and Guatemala; and as medium in Ecuador, Paraguay, Peru and Uruguay.

Overall, these findings correspond well with our results, with the exception of El Salvador and the Bolivarian Republic of Venezuela, which performed more poorly in our analysis than in both studies mentioned above.

In 2014 a global comparison between seven regions across the globe (East Asia and the Pacific, Eastern Europe and Central Asia, high-income countries, Latin America and the Caribbean, North Africa and the Middle East, South Asia and sub-Saharan Africa), found that the region Latin America and the Caribbean scored second after high-income countries in terms of the quality of vital statistics; Chile, Costa Rica, Mexico and Venezuela (Bolivarian Republic of) stood out among developing countries because they scored highly, although the registration systems in developed countries generally performed better (20).

When adding and combining more indicators for data quality, such as internal consistency and quality of reporting on age and sex, cause of death coding is shown to be an important factor in the overall performance of the registration system (20). Accurate cause of death coding is a well-known indicator of the quality of coding (18), and it can give an idea of how precisely death certificates are completed. If cause of death information is poor, this may also apply to information about place of death on the certificate. When studying place of death in relation to cause of death, as we plan to do in the next step of this project, it is important that information about both the place of death and the cause of death is as accurate as possible.

### Improving the quality of vital statistics

The majority of mortality data received were scored as being of medium quality. The study of the place of death can be useful for health care planning (1, 2), but the quality of the data needs to be strengthened and improved. The quality of vital statistics varies based on geographical and socioeconomic differences. Less affluent regions usually have worse coverage and worse quality data. These regions, due to a lack of access to health care, may also have a higher number of unregistered deaths and deaths occurring outside of hospitals, which may not be captured by our data. Cross-national comparisons are useful to identify the weaknesses in data quality in a country, but it is just as important to compare regions within a country to identify the areas with the greatest deficiencies where support for improvement is needed the most (31).

During the past 10 years, improving vital statistics has become a priority in public health (32). The relevance has already been highlighted by the *Lancet* in two series “Who counts?” (33) and “Counting births and deaths” (34). Implementing and maintaining well-functioning civil registration systems not only creates reliable sources for public health research but also creates sources for monitoring progress towards the United Nations Sustainable Development Goals and ensuring people’s access to their legal rights (35).

### Opportunities for using the data

Death certificate data have been found to be useful for public health research because they give the opportunity to study the place of death on a full-population basis rather than on limited samples (17). This provides information that can be used for improving policies at the country level.

More than 3 million deaths are documented in the combined whole-country digital data files available for the 12 countries in this study. This provides great statistical power for analyses deriving from the database, and it reflects a majority of Latin American countries. By comparing whole populations, information can be obtained about differences between health systems.

Since digital databases were easily accessible in many Latin American countries, they can be used to provide a time- and resource-saving way of collecting statistical data for research.

While the classification of place of death into private versus public institutions did not correspond to our model of classification (that is, hospital versus other health care institutions), it might provide a new dimension to investigate in future studies.

### Limitations of this study

Some limitations of our study need to be taken into account. A more comprehensive assessment has been proposed in the Lancet Global Burden of Disease study (3). In this framework, the rate of ill-defined codes is combined with several other factors that indicate data quality, so that no reference point is given for the rate of ill-defined codes as a single indicator. In an earlier study, however, Mathers et al. (18) gave reference points for classifying the rates of ill-defined codes, which we chose to apply together with the set of ill-defined codes they provided.

Additionally, the rate of mortality underreporting published by PAHO does not refer to the same year as the data files we obtained (with the exception of data for Chile). Changes may have taken place in the time between PAHO’s assessment and our analysis. In Peru, the PAHO measure was responsible for the classification of data as being low quality instead of medium quality. Because completeness ranged between 54% and 100%, not all deaths are represented in the data.

Since death certificates are completed by many people, the quality of the information can vary depending on their profession, education or how well they knew the deceased. Although we aim to estimate data quality, including the accuracy of coding, the process of completing the certificate, which our data is based upon, is difficult to verify in retrospect.

Across the 19 countries, coding for the place of death has been shown to be rather heterogeneous. This made it more challenging to fit place of death information into our categories through the reclassification or combination of categories, or both.

## Conclusions

All 19 Latin-American countries included in this study provided death certificate forms. In 18 countries, the place of death is registered on the certificate. Digital files were available for 12 countries and one region. In these, a distinction can be made between the place of death categories “home,” “hospital” or “other health care institution” and “other”. The quality of the national data sets was rated as medium to high in 10 countries. Hence, data from death certificates provide a suitable and comprehensive resource with which to research place of death and associated factors in Latin America and enable comparisons of whole populations between and across countries.

As the next step in the project, we plan to produce a descriptive analysis of the common database created from the data obtained in this study. The database makes it possible to investigate place of death and its associated factors across Latin America and identify commonalities and specificities in patterns in different countries that will provide information for health policy and priorities for future research.

**Disclaimer.** Authors hold sole responsibility for the views expressed in the manuscript, which may not necessarily reflect the opinion or policy of the *Revista Panamericana de Salud Pública/Pan American Journal of Public Health* or the Pan American Health Organization (PAHO).
